# A Common Ca^2+^-Driven Interdomain Module Governs Eukaryotic NCX Regulation

**DOI:** 10.1371/journal.pone.0039985

**Published:** 2012-06-29

**Authors:** Moshe Giladi, Yehezkel Sasson, Xianyang Fang, Reuben Hiller, Tal Buki, Yun-Xing Wang, Joel A. Hirsch, Daniel Khananshvili

**Affiliations:** 1 Department of Physiology and Pharmacology, Sackler School of Medicine, Tel-Aviv University, Ramat-Aviv, Tel-Aviv, Israel; 2 Department of Biochemistry and Molecular Biology, Faculty of Life Sciences, Tel-Aviv University, Ramat-Aviv, Tel-Aviv, Israel; 3 Protein–Nucleic Acid Interaction Section, Structural Biophysics Laboratory, National Cancer Institute at Frederick, National Institutes of Health, Frederick, Maryland, United States of America; Sackler Medical School, Tel Aviv University, Israel

## Abstract

Na^+^/Ca^2+^ exchanger (NCX) proteins mediate Ca^2+^-fluxes across the cell membrane to maintain Ca^2+^ homeostasis in many cell types. Eukaryotic NCX contains Ca^2+^-binding regulatory domains, CBD1 and CBD2. Ca^2+^ binding to a primary sensor (Ca3-Ca4 sites) on CBD1 activates mammalian NCXs, whereas CALX, a *Drosophila* NCX ortholog, displays an inhibitory response to regulatory Ca^2+^. To further elucidate the underlying regulatory mechanisms, we determined the 2.7 Å crystal structure of mammalian CBD12-E454K, a two-domain construct that retains wild-type properties. In conjunction with stopped-flow kinetics and SAXS (small-angle X-ray scattering) analyses of CBD12 mutants, we show that Ca^2+^ binding to Ca3-Ca4 sites tethers the domains via a network of interdomain salt-bridges. This Ca^2+^-driven interdomain switch controls slow dissociation of “occluded” Ca^2+^ from the primary sensor and thus dictates Ca^2+^ sensing dynamics. In the Ca^2+^-bound conformation, the interdomain angle of CBD12 is very similar in NCX and CALX, meaning that the interdomain distances cannot account for regulatory diversity in NCX and CALX. Since the two-domain interface is nearly identical among eukaryotic NCXs, including CALX, we suggest that the Ca^2+^-driven interdomain switch described here represents a general mechanism for initial conduction of regulatory signals in NCX variants.

## Introduction

The mammalian Na^+^/Ca^2+^ exchanger isoforms (NCX1–3) and their splice variants are expressed in a tissue-specific manner and catalyze electrogenic ion-exchange (3Na^+^:Ca^2+^) across the plasma membrane [1], [2], [3]. The recently determined crystal structure of an archaeal NCX from *Methanococcus jannaschii* (NCX_Mj) revealed the membrane domain architecture for this family, comprising ten transmembrane helices with a pseudo molecular dyad. Buried in the molecular center is a four-ion-binding site cluster with one site for Ca^2+^ and three for Na^+^. Two apparent passageways allow separate access for Na^+^ and Ca^2+^ ions [4]. The minimal structure supports Na^+^/Ca^2+^ exchange [4]. In contrast, the eukaryotic NCX has evolved an allosteric Ca^2+^ regulatory element encoded by a large cytosolic domain (∼500 amino acids) between transmembrane helices 5 and 6 i.e. the repeated membrane motif, designated as f-loop [5], [6], [7]. This large loop includes two Ca^2+^-binding regulatory domains (CBD1 and CBD2), connected by a short linker [6]. High-resolution NMR and crystal structures of isolated CBD1 and CBD2 domains of eukaryotic NCX revealed four Ca^2+^ sites (Ca1-Ca4) on CBD1 [8] and two Ca^2+^ sites (CaI-CaII) on CBD2 [6], [9].

Eukaryotic NCX variants differ in their response to regulatory Ca^2+^ [7], [10], [11], [12]. For example, Ca^2+^ interaction with CBD1 activates the brain (NCX1.4) and cardiac (NCX1.1) variants, whereas Ca^2+^ has no sustained effect on the kidney variant (NCX1.3) [7], [10]. In contrast, Ca^2+^ interaction with CBD1 of CALX1.1 (a *Drosophila* exchanger) inhibits the exchanger activity, whereas CALX1.2 is insensitive to regulatory Ca^2+^ [11], [12]. The Ca3-Ca4 sites of CBD1 govern Ca^2+^-dependent regulation either in NCX [13], [14] or CALX [15], [16]. Different lines of evidence suggest that CBD2 interacts with CBD1 to modify the equilibrium and kinetic properties of the primary Ca^2+^ sensor (Ca3-Ca4 sites), either in isolated CBD12 [17], [18], [19], [20] or in intact NCX [13], [14], [21]. These interdomain interactions are most prominently manifested as slow dissociation of “occluded” Ca^2+^ from Ca3-Ca4 sites of CBD12 [18], [20], which may represent the slow inactivation (I_2_ state) of intact NCX observed in electrophysiological experiments [10], [22].

Distinct techniques have been used to elucidate the structural effects of Ca^2+^ binding to isolated CBD12 [19], [21], [23]. SAXS analysis revealed that Ca^2+^ binding results in more compact conformation of CBD12 with decreased D_max_, suggesting that Ca^2+^ interaction with CBD1 changes the electrostatic potential to drive the conformational transition [19]. On the other hand, FRET studies on CBD12 revealed a small decrease in FRET upon Ca^2+^ binding to CBD1 and thus, suggest a more extended conformation of CBD12 [21]. Finally, NMR analysis of CBD12 suggested that Ca^2+^ binding to CBD1 results in rigidification of CBD12, by restricting the interdomain linker’s flexibility rather than inducing global changes in their relative reorientation [23].

Recently, high-resolution X-ray structures of CBD12 from CALX1.1 and CALX1.2 were obtained [24]. In the Ca^2+^-bound state, these structures display small differences in interdomain angles, which were suggested as the structural basis for their differential regulatory responses. According to this rationale, the interdomain angles of CBD12 might significantly differ in CALX1.1 and NCX1.4, since they exhibit diverse regulatory response to Ca^2+^. In light of these results, two critical mechanistic questions emerge. First, how precisely does Ca^2+^ binding/occlusion at the primary sensor (Ca3-Ca4 sites) couple conformational transitions in CBDs, and second, how can this coupling account for the regulatory diversity of NCX and CALX.

**Figure 1:**
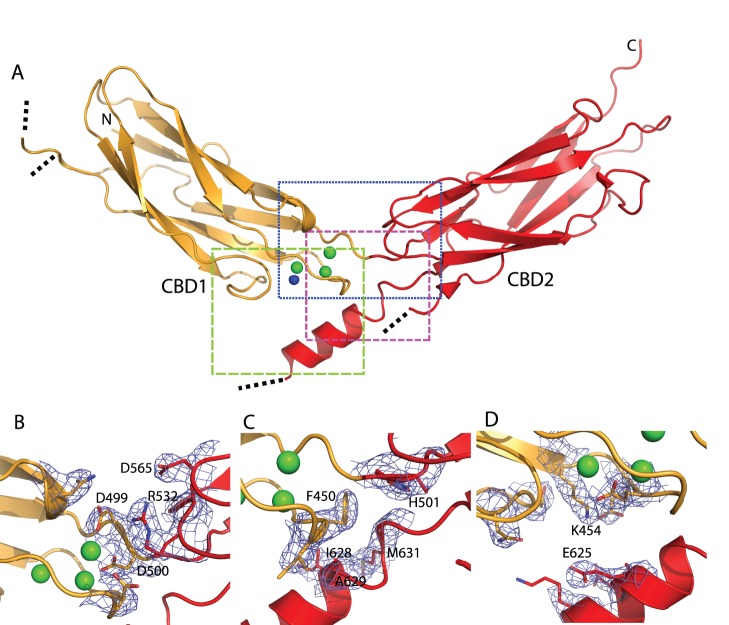
Structure of the CBD12 tandem. (A) Crystal structure of CBD12-E454K in cartoon representation. CBD1 and CBD2 are colored orange and red, respectively. The rectangles frame a zoom perspective as depicted in panels B (blue), C (magenta) and D (green). Green and blue spheres depict Ca^2+^ ions and water molecules, respectively. Dotted black lines denote electron density chain breaks in the protein. (B–D) Residues with buried surfaces in the interface are depicted as sticks, with their electron density contoured at 1.5 σ (blue mesh).

**Table 1:** Crystallographic statistics.

*Data statistics*	
Wavelength (Å)	0.976 Å
Space group	C222_1_
Unit cell parameters (Å)	*a* = 80.7, *b* = 152.5, *c* = 79.7
Total reflections	64902
Unique reflections	14002
Completeness (%)	90.0 (80.1)*
Rmerge (%)	7.9
I/σ	6.1
Resolution range (Å)	50 - 2.6
X-ray source	ESRF beamline ID29
***Refinement statistics***	
No. of reflections (working/test)	10459/898
dmin (Å)	2.68
Rwork/Rfree (%)	23.4/27.4
RMS deviation from ideality:	
Bond lengths	0.004
Bond angles	0.9
B factors (Å^2^) (rmsd of bonded atoms-main/side chain)	13.1/18.5
Average B factor (Å^2^)	84.3
No. of protein atoms/solvent	1890/15

* after anisotropic scaling.

Here, we posit that the two-domain interface initiates conduction of regulatory signals upon Ca^2+^ binding to the primary sensor. To test this working hypothesis, we crystallized and determined the crystal structure of a two-domain CBD12 tandem from the brain splice variant (NCX1.4). In conjunction with stopped-flow kinetics and SAXS analyses of relevant mutants, we demonstrate that the two-domain interface controls Ca^2+^-induced tethering of CBDs and slow dissociation of occluded Ca^2+^. In Ca^2+^-bound conformations, the interdomain angle of CBD12 is very similar for NCX1.4 and CALX1.1, meaning that the interdomain angle and/or distance between the two domains cannot account for the regulatory diversity in NCX and CALX. Therefore, Ca^2+^-binding to a high-affinity sensor (Ca3-Ca4 sites) induces a disorder-to-order transition, producing a more rigid conformation of CBD12. This interdomain switch appears to be a common module for regulation even for NCX variants exhibiting opposite responses to regulatory Ca^2+^.

**Figure 2:**
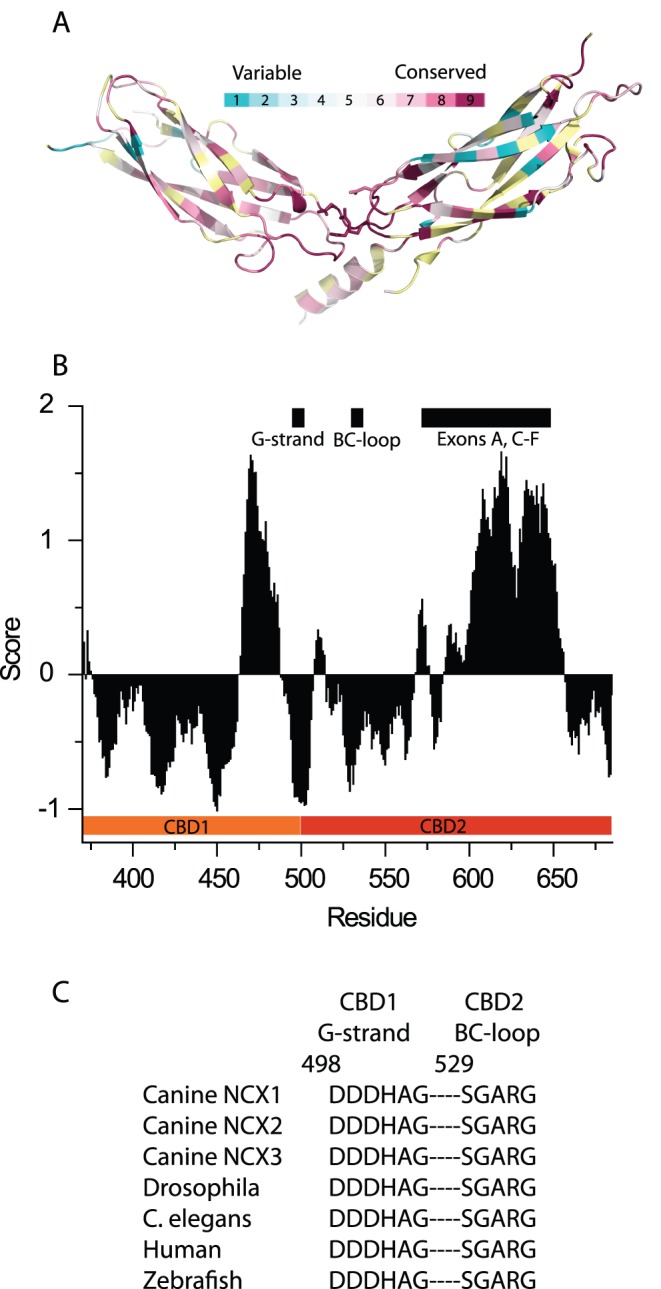
Conservation analysis of CBD domains. (A) CBD12-E454K structure colored according to the conservation score of each residue. (B) Conservation score for each residue. Negative values indicate conservation while positive values indicate variability. The sequence refers to the cardiac splice-variant, which is 35 residues longer than the brain spliced variant used in our study. (C) Conservation of interface residues located on the G-strand of CBD1 and BC-loop of CBD2.

**Figure 3:**
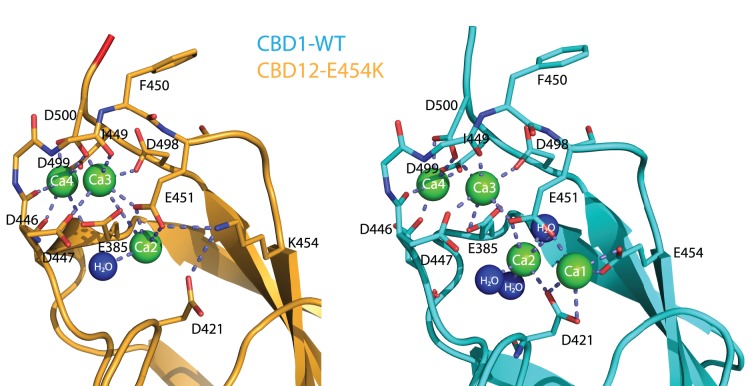
Ca^2+^ binding sites. Ca^2+^ coordination in the CBD12-E454K crystal structure (orange) and in the CBD1-WT crystal structure (cyan, PDB 2DPK). Residues coordinating Ca^2+^ are depicted as sticks.

## Results

### Structure Overview

In order to crystallize the tandem, we employed a previously characterized mutant, E454K [25]. Our rationale was three-fold. *(i)* The E454K substitution replaces the Ca^2+^ ion at Ca1 site of CBD1, thus, stabilizing the protein’s structure through a charge compensation mechanism [25]. *(ii)* The full-length NCX1-E454K mutant exhibits WT [Ca^2+^]-dependent regulation in a cellular system [25]. *(iii)* The Ca3-Ca4 sites retain their affinity for Ca^2+^ binding in an isolated CBD1-E454K [20].

The overall structure of CBD12-E454K (Figure 1A, Table 1) shows two nearly identical CBD domains, each composed of an anti-parallel β-sandwich, with root mean square deviation (r.m.s.d for 109 Cα atoms) of 1.3 Å. The domains in the tandem are also very similar to the isolated domains. The r.m.s.d between CBD1 in the tandem and isolated CBD1 (PDB 2DPK) is 0.85 Å (114 Cα atoms) while the r.m.s.d between CBD2 in the tandem and isolated CBD2 (PDB 2QVM) is 0.74 Å (114 Cα atoms).

Similar to previously published crystal structures of isolated CBD domains, the FG loops of both domains (residues 469–481 and 600–618) cannot be fully visualized due to presumed high flexibility of these areas [9]. Additionally, the N-terminal His-tag, V371 and residues 653–657 are not observed in our structure. It has been shown by NMR that the CBD2 FG-loop contains an α-helix [6], that spans residues 625 through 630 [6], [19]. Our structure now reveals the presence of an α-helix in a similar position in CBD2, spanning residues 620–629, which has side chains that contribute to the interdomain interface, described in detail below.

**Figure 4:**
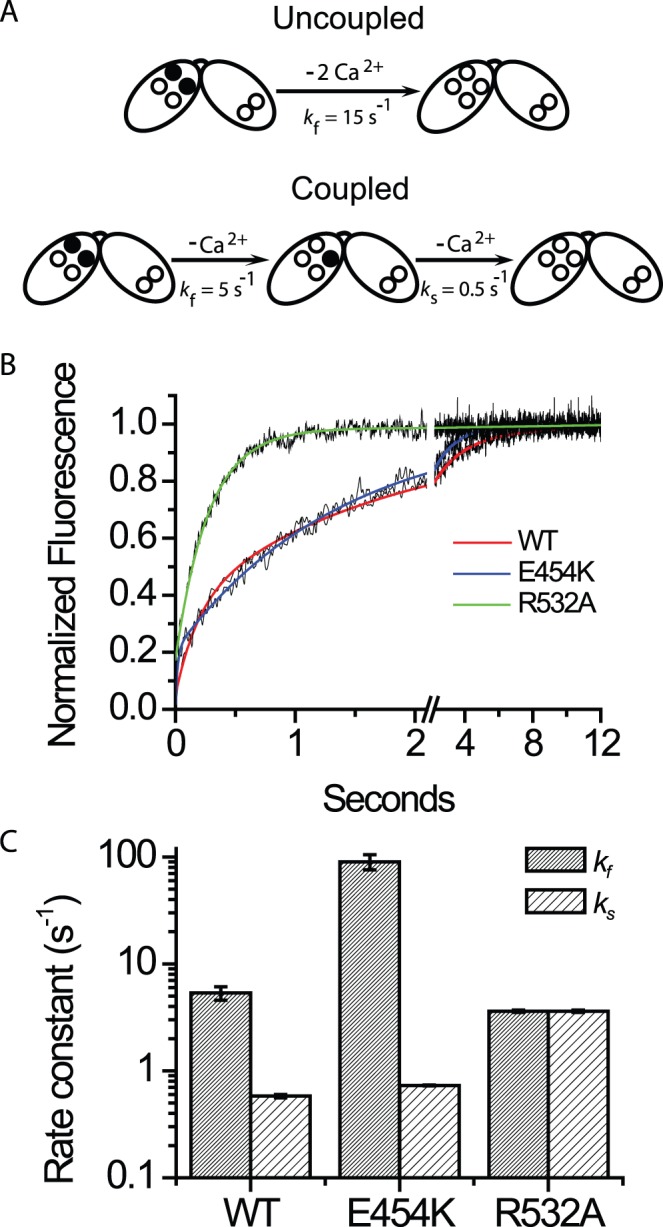
Stopped flow analysis of CBD12-E454K and CBD12-R532A . (A) Monophasic (uncoupled CBDs) and biphasic (coupled CBDs) dissociation kinetics of two Ca^2+^ ions from the Ca3-Ca4 sites, measured by stopped-flow techniques. Occupied sites are denoted by filled circles, whereas open circles represent empty sites. (B) Representative traces of Ca^2+^ dissociation kinetics from CBD12-WT, CBD12-E454K and CBD12-R532A. Ca^2+^ dissociation kinetics of CBD12-WT were fit to a double exponential curve with *k_f_* = 5.3±0.04 s^−1^ and *k_s_* = 0.57±0.001 s^−1^. The trace of CBD12-E454K was fit to a double exponential curve with *k_f_* = 52.2±1.04 s^−1^ and *k_s_* = 0.73±0.001 s^−1^. The representative trace of CBD12-R532A was fit to a single exponential curve with *k_f_* = 3.6±0.01 s^−1^. (C) Bars represent the mean ± S.E values of the “fast” phase (*k_f_*) of Ca^2+^ dissociation (n = 6) and the mean ± S.E of slow off-rates (*k_s_*) (n = 6). For CBD12-R532A, in which monophasic dissociation is observed, *k*_f_ and *k*_s_ are identical.

**Figure 5:**
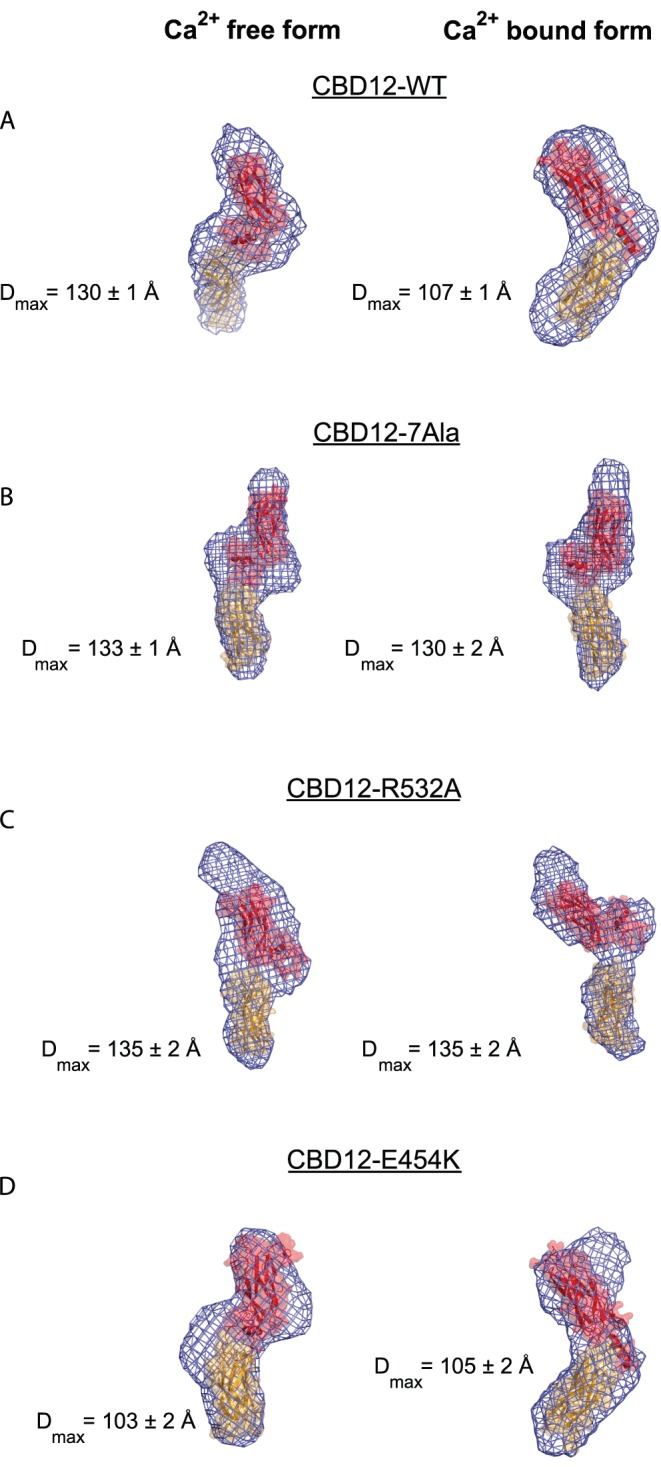
SAXS analysis of CBD12 proteins Bead model reconstruction was performed for each protein on the basis of experimental SAXS measurements. The experiments were done in the absence (left column) and presence (right column) of Ca^2+^, for the wild-type CBD12-WT (A), CBD12-7A (B) CBD12-R532A (C) and CBD12-E454K (D). The CBD1 domain (orange, PDB code: 2FWS) and the CBD2 domain (red, PDB code: 2FWU) are shown as cartoon and surface, and manually fit to the bead model shape (blue mesh), excepting panel A and D, right, where the cartoon is a depiction of the current crystal structure.

### Interdomain Interface

The interface has a buried surface area of ∼350 Å^2^. This small interface surface area is in agreement with our previous observations that isolated CBDs do not interact in solution and that a short linker is obligatory for interdomain interactions [20]. The interface involves mainly interactions of the CBD1 Ca^2+^ binding loops with the interdomain linker, the CBD2 flexible FG loop and the strictly conserved CBD2 BC loop (Figure 1). Interacting residues have low relative B-factors and are highly, if not absolutely conserved (Figure 2, S1 and Table S1), a hallmark of having an important structural or functional role.

Although more than 20 residues are buried in the interface, only a few prominent interactions between residues from both domains are observed. The interface, whose electron density is of high quality, may be subdivided into three regions, two hydrophilic (Figure 1B, D) and one hydrophobic (Figure 1C). The first hydrophilic region (Figure 1B) includes a pivotal interdomain electrostatic network centered at R532 in CBD2. This arginine takes a conformation that forms bifurcated hydrogen-bonded and non-hydrogen-bonded salt-bridges with D499, D500 in CBD1 and D565 in CBD2. D499 and D500 also participate in the coordination of the Ca3-Ca4 sites, thereby playing a direct role at the primary Ca^2+^ sensor while concomitantly stabilizing the tandem domain interface. This interfacial region is the most highly conserved among NCX isoforms and orthologs (Figure 2). The network clearly acts as the principal linchpin holding the two CBDs together.

**Table 2:** D_max_ and *k*_s_ values of CBD12 mutants.

	D_max_ (Å)		*k*_s_ (s^−1^)
	Ca^2+^-free	Ca^2+^-bound	
WT	130±1	107±1	0.58±0.02
E454K	103±2	105±2	0.73±0.007
7A	133±1	130±2	∼17 (ref 18)
R532A	135±2	135±2	3.61±0.09

Ca^2+^ -bound and -free refers to SAXS measurements performed in the presence of 10 mM CaCl_2_ or EDTA, respectively. *k*_s_ values are the mean ± SEM from 6 independent measurements derived from stopped-flow experiments and represent the slowest rate constant measured.

The hydrophobic interfacial region (Figure 1C) comprises residues from the Ca^2+^-binding EF loop of CBD1, the linker and the FG loop of CBD2. F450 serves as a core residue, forming van-der Waals interactions with H501, I628, A629, M631 and G632. It was shown that mutating residues participating in this region affect Ca^2+^ binding in CALX [24], even though they do not participate in Ca^2+^ binding directly. This finding suggests that Ca^2+^ binding affects the hydrophobic interfacial region and vice versa. Thus, this region may directly limit linker flexibility through the interaction of F450 with H501 in a Ca^2+^ dependent manner, as previously suggested by NMR data [23].

The second hydrophilic interfacial region (Figure 1D) is formed between the CBD1 CD and EF loops and the CBD2 FG loop helix. This interfacial region contains few specific interactions, although its residues are largely not surface accessible. Notably, K454 and E625 form a non-hydrogen-bonded salt-bridge (distance of 4.7 Å), a result of the mutant E454K protein used for crystallization. This salt bridge has a similar counterpart in CALX, formed between R673 and E521 [24].

**Figure 6:**
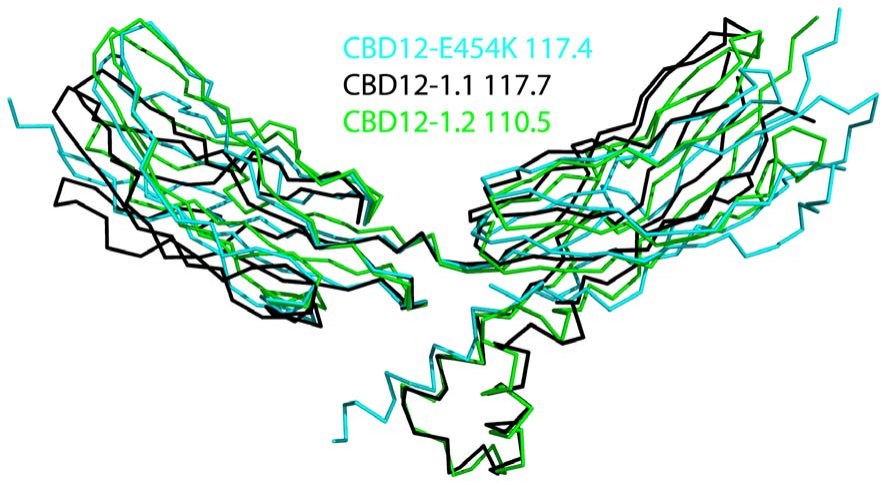
Superposition of CBD12 from NCX and CALX. The structures of CBD12-E454K from NCX1 and of CBD12-1.1 and CBD12-1.2 from CALX (PDB codes 3RB5 and 3RB7, respectively) are colored cyan, black and green, respectively. The indicated values represent the hinge angle between CBD1 and 2 as defined by Cα atoms K373, H501 and E647 (NCX CBD12-E454K) and R443, H553 and I692 (CALX CBD12-1.1 and CBD12-1.2).

### Ca^2+^ Binding Sites

In agreement with the previously published crystal structure of CBD1-E454K [25], our structure reveals three Ca^2+^ ions in CBD1 that correspond to Ca2, Ca3 and Ca4 in CBD1-WT (Figure 3). Their low solvent accessibility indicates the structural role of Ca^2+^ binding for CBD1, which loses its structural integrity in the apo-form [6], [8].

Ca2 is coordinated by E385, E451 and a water molecule, consistent with low affinity binding at this site. Residues E385, D447, I449, E451, D498 and D500 coordinate Ca3, whereas E385, D446, D447, D499 and D500 coordinate Ca4. E454K forms salt-bridges with D421 (non-hydrogen-bonded) and E451 (hydrogen-bonded), replacing Ca1 of CBD1-WT. The overall coordination is similar to that reported for isolated CBD1 [8] (Figure 3), with a few exceptions. E385 coordinates only Ca3 in isolated CBD1, while it coordinates Ca2, Ca3 and Ca4 in our structure. D421 coordinates Ca2 in CBD1, but here it only forms salt-bridge with K454, in agreement with the structure of isolated CBD1-E454K [25]. Notably, there is no indication for bound Ca^2+^ ions in CBD2 of our structure. We ascribe the lack of bound Ca^2+^ in CBD2 to crystal packing between symmetry mates. This intermolecular interaction utilizes residues that coordinate Ca^2+^ in the context of the isolated CBD2 [9] (Figure S2).

### Ca^2+^ Dissociation kinetics of CBD12 Mutants

We have previously shown that in CBD12, bi-phasic dissociation kinetics of Ca^2+^ from the Ca3-Ca4 sites are observed, with slow dissociation of one Ca^2+^ ion (*k*_s_ = 0.5 s^−1^). This sequential dissociation of Ca^2+^ is not observed either in isolated CBD1, mixture of isolated CBD1 and CBD2 or in CBD12-7A (containing additional seven alanine residues in the linker), and thus, serves as a functional hallmark of domains coupling [20] (Figure 4A). To test whether CBD12-E454K retains sequential dissociation kinetics, we performed stopped-flow assays. In agreement with the intact allosteric regulation of NCX1-E454K, sequential dissociation of Ca^2+^ is observed, although with ∼ 10 fold increase in *k*_f_ (fast phase) representing the dissociation of the first Ca^2+^ ion from Ca3-Ca4 sites of CBD12 (Figure 4B,C). To test the role of the observed electrostatic network in our structure (Figure 1B), we also tested the mutant CBD12-R532A. Strikingly, monophasic dissociation is observed, with a rate constant similar to that of isolated CBD1 [17], [20] (Figure 4B,C) despite the fact that R532 does not directly coordinate Ca^2+^ at the Ca3-Ca4 sites. Thus, the electrostatic interdomain network has a role in functional coupling of CBDs.

### SAXS Analysis of CBD12 Mutants

We utilized SAXS to detect Ca^2+^-dependent reorientation of the CBDs. The pair distance distribution function (PDDF) of CBD12-WT (Figure S3) reveals a conformational “switch” upon Ca^2+^ binding, with D_max_ = 130±1 Å and D_max_ = 107±1 Å for the Ca^2+^-free and Ca^2+^-bound forms, respectively (Figure 5A). These data are in a good agreement with previous SAXS measurements [19].

As based on the small interface surface area observed in our crystal structure and the absence of sequential dissociation kinetics [20], the insertion in CBD12-7A should abolish interaction between the two domains. Indeed, CBD12-7A lacks Ca^2+^ dependent reorientation of the CBDs (Figure 5B and Figure S3). Like CBD12-7A, CBD12-R532A does not show Ca^2+^ dependent reorientation of the CBDs (Figure 5C and Figure S3). Since CBD12-7A likely abolishes the interactions between the two domains, the similar phenotype exhibited by CBD12-R532A supports a key role for R532 in the interdomain network. Both of these mutants adopt only the extended conformations, observed for Ca^2+^-free CBD12-WT, correlating with their lack of Ca^2+^ occlusion. These results agree well with our crystallographic and biochemical data. Thus, the SAXS analysis appears to corroborate the structure-function relationship of the interdomain interface.

NCX1-E454K is regulated by Ca^2+^ as with NCX1-WT as assessed by electrophysiological studies [25]. In our SAXS analysis, however, CBD12-E454K shows no Ca^2+^ dependency for orientation of the CBDs (D_max_ = 103±2 Å and D_max_ = 105±2 Å for the Ca^2+^-free and Ca^2+^-bound forms, respectively) and maintains only the Ca^2+^-bound D_max_ of CBD12-WT according to our SAXS analysis (Figure 5D and Figure S3). Again, this result correlates well with CBD12-E454’s Ca^2+^ occlusion behavior, supporting the hypothesis that interactions relevant for Ca^2+^ occlusion exist in the Ca^2+^ bound conformation. Table 2 summarizes D_max_ and *k*_s_ values of mutants analyzed by SAXS. Overall, SAXS parameters for CBD12 and its mutants are summarized in Table S2.

## Discussion

### The Crystal Structure of Mammalian CBD12 Reveals a Ca^2+^-driven Interdomain Switch

The most important structural finding is that the two domains are tethered in a Ca^2+^-dependent manner, involving amino acids from both CBD1 and CBD2 (Figure 1). The buried R532, located in CBD2, is a central residue in the interface, tethering D565 in CBD2 and D499 and D500 in CBD1. Arginine, as opposed to lysine, appears to be required at this position due to the precise stereochemistry of the surrounding aspartates. Most importantly, the bifurcated salt-bridges between R532 and D499 and D500 support Ca^2+^ coordination at the Ca3-Ca4 sites. Therefore, Ca^2+^ binding to the Ca3-Ca4 sites couples directly to the interdomain interface to restrict the interdomain flexibility of the CBDs, as suggested by NMR (29). Consistent with this interpretation, Ca^2+^ binding to Ca3-Ca4 sites seems to be obligatory for robust interdomain interactions, since D499 and D500 are disordered in the apo-form [6], [16]. A recent NMR study revealed that Ca^2+^ binding to CBD1 restricts the flexibility of the CBD1-CBD2 linker to rigidify interdomain movements without global changes in CBDs reorientation [23]. The Ca^2+^-induced tethering of CBDs, described here, represents a new mode for ligand-induced rigidification of CBDs. Most probably, both mechanisms contribute in a cooperative way to the decoding of the regulatory message.

The CBD12-E454K mutant does not undergo global conformational changes in response to Ca^2+^ binding (Table 2), even though physiology shows NCX-E454K to be Ca^2+^ regulated [25]. This is not surprising, in light of the mutation’s stabilizing effect on the local order of CBD1 and possibly due to the salt-bridge formation with CBD2 (Figure 1D). It is possible that in the context of intact NCX, structural constraints would result in smaller or no change in D_max_. This possibility emphasizes the role of Ca^2+^-induced rigidification in allosteric regulation rather than large conformational changes. The reduced rigidity of CBD12-E454K in the absence of Ca^2+^ can be qualitatively assessed from the less-featured PDDF of its Ca^2+^-free form (Figure S3). Thus, our and NMR data point to a sequential scheme whereby Ca^2+^ occupation of Ca3-Ca4 sites orients side-chains D499 and D500 pairing with R532, and in turn restricts linker flexibility and interdomain motions in regulating NCX activity.

### The Interface Governs Dynamic Aspects of the Primary Ca^2+^ Sensor in CBD1

Our findings clearly indicate that the interface participates in slow dissociation of occluded Ca^2+^ from CBD12. Our structure shows a proximity between two charged residues in CBD2 (R532 and D565) and two Ca^2+^ coordinating residues (D499 and D500) at Ca3-Ca4 sites of CBD1. The R532 mutant of CBD12 lacks the slow Ca^2+^ dissociation (Figure 3B,C) and Ca^2+^-dependent conformational transition observed by SAXS (Fig 4C). We speculate that after dissociation of the first Ca^2+^ ion, the interdomain salt-bridges prevent complete unfolding of Ca3-Ca4 sites in CBD1 by electrostatic compensation, thereby enabling occlusion of the remaining ion. Following dissociation of the second Ca^2+^ ion, CBD1 binding sites undergo further unfolding, resulting in the apo-form orientation (D_max_ ∼ 130 Å) and no interaction of R532 with CBD1. Therefore, the R532 mutation results in “intrinsic uncoupling” of interdomain interactions, exhibiting a phenotype of non-interacting CBDs. Thus, CBD decoupling may impart decreased affinity for [Ca^2+^]-dependent activation in a NCX1-R532C mutant due to the low affinity binding of Ca^2+^ to CBD2 [14].

### Structural Comparison of the CBD12 Interface with other Proteins

Interface conservation and composition points to a general mechanism for the NCX family. Importantly, the architecture of this interface differs from the tandem C2 domains of synaptotagmin and PKC [26], implying a different mode of action. Rather, motif searches [27] of the PDB reveal a striking similarity with the cadherin extracellular domain, which bears multiple β-sandwich domains bridged by small interfaces, and which contains three Ca^2+^ sites. Cadherin studies demonstrated that Ca^2+^ rigidifies the protein [28], enabling cell-cell interactions. Furthermore, in vivo studies suggest that extracellular Ca^2+^ fluctuations may physiologically regulate cadherin activity [29], suggesting the relevance of Ca^2+^-dependent rigidification. In addition, the tandem architecture is reminiscent of the arrestin family, where tandem β sandwiches are apposed by a polar core of buried charged residues. Disruption of this polar core activates arrestin for high-affinity binding to its GPCR target [30]. These parallel actions share a common denominator of interfaces with charge interactions wherein charged ligands impart structural transitions.

### A Common Mechanism Underlies Ca^2+^-driven Interdomain Switch in the NCX Family

Recently, the crystal structures of CBD12 tandems from the two CALX splice variants (1.1 and 1.2) were determined [24]. CALX is a Na^+^/Ca^2+^ exchanger from *Drosophila* in which the binding of Ca^2+^ to CBDs inactivates the exchanger (1.1 variant) or has no effect (1.2 variant) [12]. Zheng and coworkers proposed that the stark regulatory differences between the two CALX splice variants arise from different hinge angles between the domains, thereby altering signal transmission to the membrane domain [24]. In agreement with the high degree of structural similarity between isolated CBD domains from NCX and CALX [8], [9], [15], [16], the structures of the tandem CBD12-E454K and CALX CBD12-1.1 molecules are similar (r.m.s.d of 1.86 Å for 214 Cα atoms) (Figure 6). Since the hinge angle is similar for CBD12-E454K, CALX CBD12-1.1 and CALX CBD12-1.2 (117.4°, 117.7°, and 110.5°, respectively) (Figure 6), and taking into account the existence of interdomain movements, the conformation of these domains is probably very similar in solution. The structural similarity between CBD12 from NCX and CALX (Figure 6) implies that the different responses to regulatory Ca^2+^ cannot be attributed solely to the orientation of CBDs in the CBD12 tandem. It is also possible that the conformational dynamic responses to Ca^2+^ binding differ in CBD12 of NCX and CALX, despite the similar orientation of CBDs present in these crystal structures. We hypothesize that additional structural elements in the regulatory f-loop and/or membrane domain are probably involved in specifying their differing regulatory effects. Identification of these structural elements is a crucial task for further defining the mechanism underlying Ca^2+^ regulation.

In summary, we solved the crystal structure of a two-domain tandem mutant from NCX1’s intracellular regulatory loop and found that the CBDs communicate via a complex network of electrostatic interactions at their interface. We found a correlation between the dynamic properties of CBD12 and structural data revealing Ca^2+^-driven communication between CBDs. Most importantly, occupation of Ca3-Ca4 sites by Ca^2+^ induces a disorder-to-order transition (40) due to charge neutralization and coordination, thereby constraining CBD conformational freedom, rigidifying the NCX1 f-loop, and triggering regulatory signal transmission to the membrane domain. Undoubtedly, additional structural motifs are involved in transmitting the regulatory signal in NCX variants.

## Materials and Methods

### Overexpression, Mutagenesis, and Purification of CBD12 Proteins

The DNA constructs of CBD12 (encoding residues 371–657) of canine NCX1 (accession code P23685; AD-splice variant) were cloned into pET23b vector and expressed in *E. coli* Rosetta2 (DE3) competent cells (Novagen), as described [17], [18], [20]. Mutations were introduced by QuickChange mutagenesis (Stratagene) and confirmed by sequencing. Overexpressed proteins were purified on Ni-beads followed by size exclusion chromatography (>95% purity, judged by SDS-PAGE).

### Crystallization, Data Collection and Structure Determination

Purified CBD12-E454K at a concentration of ∼30 mg/mL was prepared in buffer composed of 100 mM KCl, 10 mM CaCl_2_, 10 mM β-mercaptoethanol and 10 mM Tris-HCl at pH 7.2. Initial crystallization screens were performed at 19°C with Macrosol MD1-22 screen (Molecular Dimensions, Inc.) using the sitting drop vapor diffusion method. Initial crystals were obtained with 12% PEG-8K and 200 mM NH_4_SO_4_. Crystallization was optimized by adding 10% sucrose and lowering the PEG-8K concentration to 7%. Crystals were slowly dehydrated by raising the PEG-8K concentration in the reservoir solution by 2%/day. When the PEG-8K concentration reached 30%–32%, crystals were flash-frozen in cryo-loops. Data were collected from the flash-frozen crystals cryocooled to 110° K at beamline ID29 of the European Synchrotron Radiation Facility (Grenoble, France).

Data were processed using HKL2000 [31]. Due to anisotropic diffraction, ellipsoid truncation of the data was performed using the UCLA MBI Diffraction Anisotropy Server [32]. Resolution limits were 3.1, 2.9 and 2.68 Å along a*, b* and c* axes, respectively. This anisotropically scaled data were used for refinement of an initial model. The initial model was obtained using molecular replacement with the program PHASER [33] in the PHENIX suite [34] using a data set that diffracted to ∼3 Å. The coordinates of CBD1-E454K (PDB code 3GIN) and CBD2 (PDB code 2QVM) were used as search models. The model was refined by PHENIX [34] with multiple rounds of manual model building, performed using COOT [35]. Data and refinement statistics of the improved data set are summarized in table 1. The atomic coordinates and structure factors (PDB code 3US9) have been deposited in the Protein Data Bank, Research Collaboratory for Structural Bioinformatics, Rutgers University, New Brunswick, NJ (http://www.rcsb.org/).

### Stopped-flow Experiments

The stopped-flow assays were performed with a three-syringe/two mixer SFM-3 instrument (BioLogic, France) as described [17], [18], [20]. The data were analyzed with Bio-Kine 32 V4.45 (Bio-Logic, France). In the stopped-flow experiments, the Ca^2+^ dissociation from proteins was monitored with Quin-2 as described [17], [18], [20]. Briefly, 150 µl of 10 µM protein (pre-equilibrated with [Ca]_free_ = 5–10 µM) was mixed with 150 µl of 200 µM Quin-2 in TK-buffer.

### SAXS Data Collection, Data Analysis and Bead Model Reconstruction

SAXS data were measured at beamlines 12-ID-B and 12-ID-C of the Advanced Photon Source, Argonne National Laboratory, USA and at ID14-3 of the European Synchrotron Radiation Facility (Grenoble, France). The following samples were used for the experiments: the wild-type protein CBD12 and its mutants at three concentrations (2, 5 and 10 mg/ml) in buffers containing 20 mM Tris-HCl, pH 7.5, 100 mM KCl, 20 mM β-mercaptoethanol with10 mM EDTA or 10 mM Ca^2+^. A FoXS web-server was used for computation and fitting of SAXS profiles [36]. Further description of data collection, analysis and bead model reconstitution is described in the Information S1.

### Conservation Analysis of CBD12

The Ensembl server [37] was used to search for canine cardiac NCX orthologs, which is the longest splice variant containing an additional 35 residues in CBD2. These sequences were used to generate a multiple sequence alignment using Clustalw [38]. The resulting alignment was used as input for Consurf [39], [40], [41], which outputs a conservation score for each residue.

## Supporting Information

Figure S1 **Multiple Sequence Alignment of CBD12 orthologs.** Numbering according to canine NCX1 cardiac splice variant.
(PDF)

Figure S2 **Crystal packing involves CBD2 Ca^2+^ binding sites.** (A) Interaction between symmetry mates in the CBD12-E454K crystal. (B) Salt-bridges are formed between symmetry mates, involving residues that participate in coordination of Ca^2+^ in isolated CBD2 (E516, K585).
(EPS)

Figure S3 **SAXS analysis.** Comparison of SAXS scattering curves (left column) and normalized pair distance distribution functions (PDDF) (right column) for the wild type CBD12 (A, B), CBD12-7Ala (C, D) CBD12-R532A (E, F) and CBD12-E454K (G, H), respectively, in the absence (black) or presence of Ca^2+^ (red). The scattering profiles were normalized and superimposed against the first data points.
(EPS)

Table S1 **CBD12 Interface Conservation.**
(DOCX)

Table S2 **Overall SAXS parameters for CBD12 and its mutants.** *R*_g_, *D*_max_, and NSD are, respectively, the radius of gyration derived from Guinier plotting, maximum inter-atomic dimension, normalized shape discrepancy for DAMMIN calculation. Note that NSD scores for all bead models are less than 0.8, indicating high convergence of the bead model calculations.
(DOCX)

Information S1 **Experimental procedures.**
(DOCX)
