# Supplementary material for: A Common Ca2+-Driven Interdomain Module Governs Eukaryotic NCX Regulation
Source: PLoS One. 2012 Jun 29;7(6):e39985. doi: 10.1371/journal.pone.0039985 (PMC3386913; doi:10.1371/journal.pone.0039985)
Supplement: Figure S1 — Multiple Sequence Alignment of CBD12 orthologs. Numbering according to canine NCX1 cardiac splice variant. (PDF) [file pone.0039985.s001.pdf]

Figure S1

```
                                  371
Canine_NCX1                       VSKIFFEQGTYQCLENCGTVALTIIRRGGDLT--NTVFVDFRTEDGTANA
Medaka_NCX                        TIYLQFDPSHYQCFENCGSLKLSVSRHGGESG--CTVKVDYRTEDGTANA
Tetraodon_NCX                     TIYLQFEPSHYQCFENCGSLKLSVSRYGGESG--CTVKVDYRTEDATASA
Fugu_NCX                          TIYLQFDPSHYQCFENCGSLKLSVSRHGGESG--CTVKVDYRTEDATASA
Anole_Lizard_NCX                  VTKVGFEPSHYQCFENCGSVVLTVARRGGDPGR-VTLRVDFRTEDGTANA
Human_NCX1                        VSKIFFEQGTYQCLENCGTVALTIIRRGGDLT--NTVFVDFRTEDGTANA
Rat_NCX                           VSKVFFEQGTYQCLENCGTVALTIIRRGGDLT--NTVFVDFRTEDGTANA
Megabat_NCX                       VSKIFFEQGTYQCLENCGTVALTIIRRGGDLT--NTVFVDFRTEDGTANA
Tarsier_NCX                       VSKIFFEQGTYQCLENCGTVALTIIRRGGDLT--NTVFVDFRTEDGTANA
Alpaca_NCX                        VSKIFFEQGTYQCLENCGTVALTIIRRGGDLT--NTVFVDFRTEDGTANA
Cow_NCX                           VSKIFFEQGTYQCLENCGTVALTIIRRGGDLT--NTVFVDFRTEDGTANA
Dolphin_NCX                       VSKIFFEQGTYQCLENCGTVALTIIRRGGDLT--NTVFVDFRTEDGTANA
Chimpanze_NCX                     VSKIFFEQGTYQCLENCGTVALTIIRRGGDLT--NTVFVDFRTEDGTANA
Gorilla_NCX                       VSKIFFEQGTYQCLENCGTVALTIIRRGGDLT--NTVFVDFRTEDGTANA
Orangutan_NCX                     VSKIFFEQGTYQCLENCGTVALTIIRRGGDLT--NTVFVDFRTEDGTANA
Gibbon_NCX                        VSKIFFEQGTYQCLENCGTVALTIIRRGGDLT--NTVFVDFRTEDGTANA
Macaque_NCX                       VSKIFFEQGTYQCLENCGTVALTIIRRGGDLT--NTVFVDFRTEDGTANA
Marmoset_NCX                      VSKIFFEQGTYQCLENCGTVALTIIRRGGDLT--NTVFVDFRTEDGTANA
Guinea_pig_NCX                    VSKIFFEQGTYQCLENCGTVALTIIRRGGDLT--NTVFVDFRTEDGTANA
Pika_NCX                          VSKIFFEQGTYQCLENCGTVALTIIRRG-DLT--NTVFVDFRTEDGTANA
Bushbaby_NCX                      VSKIFFEQGTYQCLENCGTVALTIIRRGGDLT--NTVFVDFRTEDGTANA
Microbat_NCX                      VSKIFFEQGTYQCLENCGTVALTIIRRGGDLT--NTVFVDFRTEDGTANA
Sloth_NCX                         VSKIFFEQGTYQCLENCGTVALTIFRRGGDLT--NTVFVDFRTEDGTANA
Hyrax_NCX                         VSKIFFEQGTYQCLN-CGTVALTIIRRGGDLT--NTVFVDFRTEDGTANA
Opossum_NCX                       VSKIYFEQGAYQCLENCGTVALNIIRRGGDLS--NTVFVDFRTEDGTANA
Platypus_NCX                      VSKIYFEQGTYQCLENCGTVALTIVRRGGDLT--NTVYVDFRTEDGTANA
Mouse_NCX                         VSKIFFEQGTYQCLENCGTVALTIMRRGGDLS--TTVFVDFRTEDGTANA
Kangaroo_rat_NCX                  VSKVFFEQGSYQCLENCGTVSLTIIRQGGNLA--NTVHVDFRTEDGTANA
Turkey_NCX                        ISKLYFEQGTYQCLENCGTVALTIIRRGGDLT--NTVYVDFRTEDGTANA
Zebra_finch_NCX                   VSKLYFEQGTYQCLENCGTVALTIVRRGGDLT--NTVYVDFRTEDGTANA
Chicken_NCX                       ISKLYFEQGTYQCLENCGTVALTIIRRGGDLT--NTVYVDFRTEDGTANA
Xenopus_tropicalis_NCX            VSKIYFEQATYQCLENCGTVALTIVRRGGDLT--NTVYVDFRTEDGSANA
Stickleback_NCX                   STKVFFDPCTYQCLENCGTVALNVVRRGGDPT--SAISVDYRTEDGTANA
Zebrafish_NCX                     SSKIFFDPGTYQCLENCGTVALNVVRRGGDLT--STVSVEYRTEDGTANA
C.elegans_NCX                     MATVEFDPPHYTCLENVGDVYLTVKCDRGSVPEDTTVTVHYRTIADTAQA
C.intestinalis_NCX                VAKIYFEPAEYKVMENCGVAELQVVRTGGDLN--TTLYVDYQTEDGTANA
C.savigny_NCX                     VAKIYFDPAEYKVMENCGVAQLQVVRTGGDLN--TTLYVDYQTEDGTANA
drosophila_NCX                    PIRMYFEPGHYTVMENCGEFEVRVVRRG-DIS--TYASVEYETQDGTASA
                                     : *:   *  ::  *   : :     .        *.:.*   :*.*

                                  419
Canine_NCX1                       GSDYEFTEGTVVFKPGETQKEIRVGIIDDDIFEEDENFLVHLSNVKVSSE
Medaka_NCX                        GSDYEFAEGTLVFKPGETTKEFTVGVIDDDIFEEDEHFYVRLSNPRIVHR
Tetraodon_NCX                     GSDYEFAEGTLVFKPGETTKEFTVGIIDDDIFEEDEHFYVRLSNPRVAHR
Fugu_NCX                          GSDYEFAEGTLVFKPGETTKEFTVGIIDDDIFEEDEHFYVRLSNPRVAH-
Anole_Lizard_NCX                  GSDYEFAEGTLLFKPGETHKEVRVGIIDDDIFEEDEYFYVHLSNVRAAWS
Human_NCX1                        GSDYEFTEGTVVFKPGDTQKEIRVGIIDDDIFEEDENFLVHLSNVKVSSE
Rat_NCX                           GSDYEFTEGTVIFKPGETQKEIRVGIIDDDIFEEDENFLVHLSNVRVSSE
Megabat_NCX                       GSDYEFTEGTVVFKPGETQKEIRVGIIDDDIFEEDENFLVHLSNVKVSSE
Tarsier_NCX                       GSDYEFTEGTVVFKPGETQKEIRVGIIDDDIFEEDENFLVHLSNVKVSSE
Alpaca_NCX                        GSDYEFTEGTVVFKPGETQKEIRVGIIDDDIFEEDENFLVHLSNVKVSAE
Cow_NCX                           GSDYEFTEGTVVFKPGETQKEIRVGIIDDDIFEEDENFLVHLSNVKVSLE
Dolphin_NCX                       GSDYEFTEGTVVFKPGETQKEIRVGIIDDDIFEEDENFLVHLSNVKVSSE
Chimpanze_NCX                     GSDYEFTEGTVVFKPGETQKEIRVGIIDDDIFEEDENFLVHLSNVKVSSE
Gorilla_NCX                       GSDYEFTEGTVVFKPGETQKEIRVGIIDDDIFEEDENFLVHLSNVKVSSE
Orangutan_NCX                     GSDYEFTEGTVVFKPGETQKEIRVGIIDDDIFEEDENFLVHLSNVKVSSE
Gibbon_NCX                        GSDYEFTEGTVVFKPGETQKEIRVGIIDDDIFEEDENFLVHLSNVKVSSE
Macaque_NCX                       GSDYEFTEGTVVFKPGETQKEIRVGIIDDDIFEEDENFLVHLSNVKVSSE
Marmoset_NCX                      GSDYEFTEGTVVFKPGETQKEIRVGIIDDDIFEEDENFLVHLSNVKVSSE
Guinea_pig_NCX                    GSDYEFTEGTVVFKPGETQKEIRVGIIDDDIFEEDENFLVHLSNVKVSSE
Pika_NCX                          GSDYEFTEGTVVFKPGETQKEIRVGIIDDDIFEEDENFLVHLSNVKVSLE
Bushbaby_NCX                      GSDYEFTEGTVVFKPGETQKEIRVGIIDDDIFEEDENFLVHLSNVKVSSE
Microbat_NCX                      GSDYEFTEGTVVFKPGETQKEIRVGIIDDDIFEEDENFLVHLSNVKVSSE
Sloth_NCX                         GSDYEFTEGTVVFKPGETQKEIRVGIIDDDIFEEDENFLVHLSNVKVSSE
Hyrax_NCX                         GSDYEFTEGTVVFKPGETQKEIRVGIIDDDIFEEDENFLVHLSNVKVSSD
Opossum_NCX                       GSDYEFTEGTVVFKPGESQKEIRVGIIDDDIFEEDENFHVHLSNVKVTSE
Platypus_NCX                      GSDYEFTEGTVAFKPGETQKEIRVGIIDDDIFEEDENFLVHLSNVKVTAE
Mouse_NCX                         GSDYEFTEGTVIFKPGETQKEIRVGIIDDDIFEEDENFLVHLSNVRVSSD
Kangaroo_rat_NCX                  GSDYEYTEGTVVFKPGETQKEIRVGIIDDDIFEEDENFLVHLSNLKVFTD
Turkey_NCX                        GSDYEFTEGTVVFKPGETQKEIRVGIIDDDIFEEDENFLVHLSNVRVSTE
Zebra_finch_NCX                   GSDYEFTEGTVVFKPGETQKEIRVGIIDDDIFEEDENFLVHLSNVRVSTE
```

```
Chicken_NCX                 GSDYEFTEGTVVFKPGETQKEIRVGIIDDDIFEEDENFLVHLSNVRVSTE
Xenopus_tropicalis_NCX      GSDYEFTEGTIIFKPGETQKELRVGIIDDDIFEEDENFLVHLSNVRVNAE
Stickleback_NCX             GSDYRFTEGTIVFIAGETEKELRIDIIDDDIFEEDEHFLVHLSNVKVISE
Zebrafish_NCX               GSDYQFTEGVIIFKPGETEKEIRVDIIDDDIFEEDEHFLVHLSNVKVISE
C.elegans_NCX               ESDFVHTEGTITFEPGQTEQKIKVGIVDNDIYEDDEQFMVRLSQVRAFRS
C.intestinalis_NCX          GSDFEHAEDTIVFKPGETTKTIAITIMDDDIFEEDEYFRVKLCNVRSGDA
C.savigny_NCX               GSDFVHAEDTLVFKPGETTRTIDIVIMDDDIFEEDEYFRVKLCNVRSGDA
drosophila_NCX              GTDFVGRKGLLSFPPGVDEQRFRIEVIDDDVFEEDECFYIRLFNPSEG--
                             :*:   :. : * .*   : . : ::*:*::*:** * ::* :

                          469
Canine_NCX1                 AS----EDGILEAN--------HVSALACLGSPSTATVTIFDDDHAGIFT
Medaka_NCX                  AEVSILEPSSITSSNSMVGISSHIPPKAALGKDPVATVTIYDDDHAGIFT
Tetraodon_NCX               AEVSILEPNSITSS----------PPEAALGKAHTATVTIYDDDHAGIFT
Fugu_NCX                    ------SNSTVGTS-------SHVPPKAALGKDHTATVTIYDDDHAGIFT
Anole_Lizard_NCX            EP----GPE--------------PPPKACLGPSKMATVTIFDDDHAGIFT
Human_NCX1                  AS----EDGILEAN--------HVSTLACLGSPSTATVTIFDDDHAGIFT
Rat_NCX                     VS----EDGILDSN--------HVSAIACLGSPNTATITIFDDDHAGIFT
Megabat_NCX                 AS----EDGILEAN--------HVSSLACLGSPSTATVTIFDDDHAGIFT
Tarsier_NCX                 AS----EDGILEAN--------HVSTLACLGSPSTATVTIFDDDHAGIFT
Alpaca_NCX                  AS----EDGILEAN--------HVSTLACLGSPSTATVTIFDDDHAGIFT
Cow_NCX                     AS----EDGILEAS--------HVSTLACLGSPSTATVTIFDDDHAGIFT
Dolphin_NCX                 AS----EDGILEAN--------HVSTLACLGSPSTATVTIFDDDHAGIFT
Chimpanze_NCX               AS----EDGILEAN--------HVSTLACLGSPSTATVTIFDDDHAGIFT
Gorilla_NCX                 AS----EDGILEAN--------HVSTLACLGSPSTATVTIFDDDHAGIFT
Orangutan_NCX               AS----EDGILEAN--------HVSTLACLGSPSTATVTIFDDDHAGIFT
Gibbon_NCX                  AS----EDGILEAN--------HVSALACLGSPSTATVTIFDDDHAGIFT
Macaque_NCX                 AS----EDGILEAN--------HVSTLACLGSPSTATVTIFDDDHAGIFT
Marmoset_NCX                AS----EDGILEAN--------HVSTLACLGSPSTATVTIFDDDHAGIFT
Guinea_pig_NCX              AS----EDGILEAN--------HISTLACLGSPSTATVTIFDDDHAGIFT
Pika_NCX                    TS----EDGILEAN--------HVSTFACLGSPCTATVTIFDDDHAGIFT
Bushbaby_NCX                AS----EDGILEAN--------HVSTLACLGSPSTATVTIFDDDHAGIFT
Microbat_NCX                AS----EDGILEAN--------HVSTLACLGSPSTATVTIFDDDHAGIFT
Sloth_NCX                   AS----EDGILEAN--------HVSTLACLGSPSTATVTIFDDDHAGIFT
Hyrax_NCX                   PS----EDGILEAN--------HMSTLACLGSPATATVTIFDDDHAGIFT
Opossum_NCX                 AL----EDGILEAN--------HIASLACLGSPSTATVTIFDDDHAGIFT
Platypus_NCX                TS----EDGIREAN--------HVATLACLGSPSTATVTIFDDDHAGIFT
Mouse_NCX                   VS----EDGILESN--------HASSIACLGSPSTATITIFDDDHAGIFT
Kangaroo_rat_NCX            TQ----EDTILEAN--------HVATLACIGSPSTATITIFDDDHAGIFT
Turkey_NCX                  A-----DEGILEAS--------RVSTLACLGSPSTATVTIFDDDHAGIFT
Zebra_finch_NCX             AS----DEGVLEAS--------RVSTLACLGSPSTATVTIFDDDHAGIFT
Chicken_NCX                 A-----DEGILEAS--------RVSTLACLGSPSTATVTIFDDDHAGIFT
Xenopus_tropicalis_NCX      N-----TEANLESN--------HVTPLACLGATCTATVTIFDDDHAGIFT
Stickleback_NCX             GA----GWDGLKAN--------HHDPLASLGLPCSATVTIFDDDHAGIFT
Zebrafish_NCX               GA----NNGNPGTN--------HVDALAGLGLPSTATVTIFDDDHAGIFL
C.elegans_NCX               EH--------------------FSSVPARLGLAATATVIIVDDDHAGSFG
C.intestinalis_NCX          DG-------MFDTK-------ANSTQVARLEPPAVATVVILDDDHAGVFS
C.savigny_NCX               DG-------MFDTK-------GNSMQVARLEPPAVATVVILDDDHAGVFS
drosophila_NCX              ---------------------------VKLAVPMIATVMILDDDHAGIFA
                                                       . :     **: * ****** *

                          507
Canine_NCX1                 FEEPVTHVSESIGIMEVKVLRTSGARGNVIVPYKTIEGTARGGGEDFEDT
Medaka_NCX                  FESKSMRVSESVGNMQVKVHRTSGARGKVAVPYHTVEGTAKAG-EDYEDV
Tetraodon_NCX               FESDSTKVSESIGNMQVKVHRTSGARGKVAIPYHTVEATAKAG-EDYEEV
Fugu_NCX                    FESDTTKVSESIGNMQVKVHRTSGARGKVAVPYHTVEGTAKAG-EDYEEV
Anole_Lizard_NCX            FEGASMRVSESVGAVRIKVLRTSGARGRVAIPFHTIEGTAKAG-EDYEEV
Human_NCX1                  FEEPVTHVSESIGIMEVKVLRTSGARGNVIVPYKTIEGTARGGGEDFEDT
Rat_NCX                     FEEPVTHVSESIGIMEVKVLRTSGARGNVIIPYKTIEGTARGGGEDFEDT
Megabat_NCX                 FEEPVTHVSESIGIMEVKVLRTSGARGNVIVPYKTIEGTARGGGEDFEDT
Tarsier_NCX                 FEEPVTHVSESIGIMEVKVLRTSGARGNVIVPYKTIEGTARGGGEDFEDT
Alpaca_NCX                  FEEPVTHVSESIGIMEVKVLRTSGARGNVIVPYRTIEGTARGGGEDFEDT
Cow_NCX                     FEEPVTHVSESIGIMEVKVLRTSGARGNVIVPYKTIEGTARGGGEDFEDT
Dolphin_NCX                 FEEPVTHVSESIGIMEVKVLRTSGARGNVIVPYKTIEGTARGGGEDFEDT
Chimpanze_NCX               FEEPVTHVSESIGIMEVKVLRTSGARGNVIVPYKTIEGTARGGGEDFEDT
Gorilla_NCX                 FEEPVTHVSESIGIMEVKVLRTSGARGNVIVPYKTIEGTARGGGEDFEDT
Orangutan_NCX               FEEPVTHVSESIGIMEVKVLRTSGARGNVIVPYKTIEGTARGGGEDFEDT
Gibbon_NCX                  FEEPVTHVSESIGIMEVKVLRTSGARGNVIIPYKTIEGTARGGGEDFEDT
Macaque_NCX                 FEEPVTHVSESIGIMEVKVLRTSGARGNVIVPYKTIEGTARGGGEDFEDT
Marmoset_NCX                FEEPVTHVSESIGIMEVKVLRTSGARGNVIVPYKTVEGTARGGGEDFEDT
Guinea_pig_NCX              FEEPVTHVSESIGIMEVKVLRTSGARGNVIVPYKTIEGTARGGGEDFEDT
Pika_NCX                    FEEAVTHVSESIGIMEVKVLRTSGARGNVIVPYKTIEGTARGGGEDFEDT
```

```
Bushbaby_NCX             FEEPVTHVSESIGIMEVKVLRTSGARGNVIVPYKTIEGTARGGGEDFEDT
Microbat_NCX             FEEPVTHVSESVGIMEVKVLRTSGARGNVIVPYKTIEGTARGGGEDFEDT
Sloth_NCX                FEEAVTHVSESIGIMEVKVLRTSGARGNVIVPYKTIEGTARGGGEDFEDT
Hyrax_NCX                FEEPVTHVSESIGIMEVKVLRTSGARGNVIVPYKTIEGTARGGGEDFEDT
Opossum_NCX              FEEPVTHVSESVGIMEVKVLRTSGARGSVIVPYKTIEGSARGGGEDFEDT
Platypus_NCX             FEEPVTHVSESVGVMEVKVLRTSGARGNVIVPYKTIEGSARGGGEDFEDT
Mouse_NCX                FEEPVTHVSESIGIMEVKVLRTSGARGNVIIPYKTIEGTARGGGEDFEDT
Kangaroo_rat_NCX         FEESVLHVSESIGTMEVKVLRTSGARGNVIVPYKTIEGTAKGGGEDFEDT
Turkey_NCX               FEEPVTHVSESVGTMEVKVLRTSGARGNVIVPYKTIEGSAKGGGEDFEDT
Zebra_finch_NCX          FEEPVTHISESVGTMEVKVLRTSGARGNVIVPYKTIEGTAKGGGEDFEDT
Chicken_NCX              FEEPVTHVSESVGTMEVKVLRTSGARGNVIVPYKTIEGSAKGGGEDFEDT
Xenopus_tropicalis_NCX   FEETVTHVSESVGIMEVKVLRTSGARGTVIVPYKTVEGTAKGGGEDFEDT
Stickleback_NCX          FENPALTVSESVGVMEARVVRTSGARGVVAVPYKTREGTAKGGGEDFEDT
Zebrafish_NCX            FEEPIVHISESIGMMEVKVVRTSGARGVVVIPYKTIEGTAKGGGEDFEDT
C.elegans_NCX            FLSEKFKCTESCGSFVAEVIRSRGARGKVSIPYKTVDGAAKSP-QDYEHQ
C.intestinalis_NCX       FPEPTITVSEGVGVLKVEVQRNSGARGRITVPYKTVSGTAKGGGEDYIDA
C.savigny_NCX            FPEPSITVSEGVGVLKIEVQRNSGARGRIIVPYKTVNGTAKGGGEDFIDT
drosophila_NCX           FTDSVFEITESVGRFELKVMRYSGARGTVIVPYWTENDTATES-KDYEGA
                         *       :*. * .  .* *  **** : :*: * . :*    :*:

                       557
Canine_NCX1              CGELEFQNDEIVKTISVKVIDDEEYEKNKTFFLEIGEPRLVEMSEKKALL
Medaka_NCX               SGKLEFQNDETMKLLNVKIIDDEEYEKNKIFTVVLEEPILLEVGQRHDSQ
Tetraodon_NCX            SGKLEFQNDETMKLLNVKIIDDEEYEKNKTFTIVLEEPILLEVGQKHAST
Fugu_NCX                 SGKLEFQNDETMKLLNVKIIDDEEYEKNKTFTIVLEEPILLEVGQKHGER
Anole_Lizard_NCX         AGKVEFQNDETMKYIEIKIIDDEEYEKNKNFHLELGPPVLLDTGPRHGII
Human_NCX1               CGELEFQNDEIVKIITIRIFDREEYEKECSFSLVLEEPKWIR-RGMK---
Rat_NCX                  CGELEFQNDEIVKIITIRIFDREEYEKECSFSLVLEEPKWIR-RGMKALL
Megabat_NCX              CGELEFQNDEIVKTISVKVIDDEEYEKNKTFFLEIGEPRLVEMSEKKALL
Tarsier_NCX              CGELEFQNDEIVKTISVKVIDDEEYEKNKTFFLEIGEPRLVEMSEKKALL
Alpaca_NCX               CGELEFQNDEIVKTISVKVIDDEEYEKNKTFFLEIGEPRLVEMSEKKALL
Cow_NCX                  CGELEFQNDEIVKTISVKVIDDEEYEKNKTFFLEIGEPRLVEMSEKKALL
Dolphin_NCX              CGELEFQNDEIVKTISVKVIDDEEYEKNKTFLLEIGEPRLVEMSEKKALL
Chimpanze_NCX            CGELEFQNDEIVKTISVKVIDDEEYEKNKTFFLEIGEPRLVEMSEKKALL
Gorilla_NCX              CGELEFQNDEIVVLYYMCVC---VLAFSTLRSLALSSKRLVQMSESYTLL
Orangutan_NCX            CGELEFQNDEIVKTISVKVIDDEEYEKNKTFFLEIGEPRLVEMSEKKALL
Gibbon_NCX               CGELEFQNDEIVKTISVKVIDDEEYEKNKTFFLEIGEPRLVEMSEKKALL
Macaque_NCX              CGELEFQNDEIVKTISVKVIDDEEYEKNKTFFLEIGEPRLVEMSEKKEIV
Marmoset_NCX             CGELEFQNDEIVKTISVKVIDDEEYEKNKTFFLEIGEPRLVEMSEKKEIV
Guinea_pig_NCX           CGELEFQNDEIVKTISVKVIDDEEYEKNKTFFLEIGEPRLVEMSEKKALL
Pika_NCX                 CGELEFQNDEIVKTISVKVIDDEEYEKNKTFFLEIGEPRLVEMSEKKALL
Bushbaby_NCX             CGELEFQNDEIVKTISVKVIDDEEYEKNKTFFLEIGEPRLVEMSEKKALL
Microbat_NCX             CGELEFQNDEIVKTISVKVIDDEEYEKNKTFFLEIGEPRLVEMSEKKALL
Sloth_NCX                WGELEFQNDEIVKTVSVKVIDDEEYEKNKTFFLEIGEPRLVEMSEKKALL
Hyrax_NCX                CGELEFQNDEIVKTISVKVIDDEEYEKNKTFFLEIGEPRLVEMSEKKXXX
Opossum_NCX              CGELEFQNDEILKTISVKVIDDEEYEKNKTFFLEIGEPRLVEMSEKKALL
Platypus_NCX             CGELEFQNDEIVKTISVKVIDDEEYEKNKTFFLEIGEPRLVEMSEKK---
Mouse_NCX                CGELEFQNDEIVKTISVKVIDDEEYEKNKTFFIEIGEPRLVEMSEKKALL
Kangaroo_rat_NCX         CGQLEFQNDEIIKTISVKVIDDEEYET-KTFFLEN-EPRLVEMSEKKALL
Turkey_NCX               CGELEFQNDEIVKTISIKVIDDEEYEKNKTFYLEIGEPRLVEMSEKKALL
Zebra_finch_NCX          CGELEFQNDEIVKTISIKVIDDEEYEKNKTFYLEIGEPRLVEMSEKKALL
Chicken_NCX              CGELEFQNDEIVKFITLRILDREEYEKECSFFLVLGDP------------
Xenopus_tropicalis_NCX   CGQLEFQNDEIVKIISVKIIDDEEYEKNKTFFLEVGEPRLVEMSEKKALL
Stickleback_NCX          HGVLEFENDEIFKTIQINIIDDEEYEKNKNFFLEIGEPQLLEMSERKAVL
Zebrafish_NCX            HGVLEFQNDEISKTIQINIIDDEEYEKNKNFFLEIGEPQLVEMSERKAML
C.elegans_NCX            EGVLKFADEQSKAEIYIPIVNDDEEYEKHEDFYIELGEPIW----------
C.intestinalis_NCX       IGELDFDNDETTKCINVTIIDDEEYEKNKSFFVELSQPKLYSDKDSGLRR
C.savigny_NCX            IGELEFDNDETTKHISVPIVDDEEYEKNKSFFVELSQPKFYSDKDSGLTV
drosophila_NCX           RGELVFENNESEKFIDLFILEESSYEKDVSFKVHIGEPRLAPDD------
                          * : * :::      : :             :

                       607
Canine_NCX1              LNELGG-FTITGKYLYGQ-PVFRKVHAREHPIPSTVITIAEEYDDKQPLT
Medaka_NCX               TNSLCP-HSFTALNLWGR-----TCNYRRLPCHSKLFRCAGDTNDNKTAV
Tetraodon_NCX            ---------------------------------------AGDTNDNKTSV
Fugu_NCX                 RRSAAI-LLFVSR------------YKTPVVCVCVCVSTAGDTNDNKATV
Anole_Lizard_NCX         IP-------------------------------------GDSNENRLPE
Human_NCX1               ----GG-FTIT-----------------------------DEYDDKQPLT
Rat_NCX                  LNELGG-FTLTGKKMYGQ-PVFRKVHARDHPIPSTVISISEEYDDKQPLT
Megabat_NCX              LNELGG-FTITGKYLYGQ-PVFRKVHAREHPIPSTVITIAEECDDKQPLT
Tarsier_NCX              LNELGG-FTITGKYLYGQ-RVFRKVHAREHPIPSTVITIAEEYDDKQPLS
Alpaca_NCX               LNELGG-FTITGKYLYGQ-PVFRKVHARERPIPSSIITIAEECDDKQ-LT
```

```
Cow_NCX                     LNELGG-FTITGKYLYGQ-PVFRKVHAREHPLPSTIITIADEYDDKQPLT
Dolphin_NCX                 LNELGG-FTITGKYLYGQ-PVFRKVHAREHPIPSTIITIA-EYDDKQPLT
Chimpanze_NCX               LNELGG-FTITGKYLFGQ-PVFRKVHAREHPILSTVITIADEYDDKQPLT
Gorilla_NCX                 LNELGG-FTITGKYLFGQ-PVFRKVHAREHPILSTVITIADEYDDKQPLT
Orangutan_NCX               LNELGG-FTITGKYLFGQ-PVFRKVHAREHPILSTVITIADEYDDKQPLT
Gibbon_NCX                  LNELGG-FTITGKYLFGQ-PVFRKVHAREHPILSTVITIADEYDDKQPLT
Macaque_NCX                 LFSLRG-FTITGKYLYGQ-PVFRKVHAREHPILSTVITITDEYDDKQPLT
Marmoset_NCX                LFSLRG-FTITGKYLYGQ-PVFRKVHAREHPIPSTVITIADEYDDKQPLT
Guinea_pig_NCX              LNELVGGFTITGKHLYGQ-PVLRKVHARDHPIPSTVITIADEYDDKQPLT
Pika_NCX                    LNELGG-FTITGKYLCGQ-PVFRKVHARDHPIPSTVITIADEYDDRQPLT
Bushbaby_NCX                LNELGG-FTITGKCLYGE-PVFRKVHAREHPI-STIITFSEEYDDKQPLT
Microbat_NCX                LNELGG-FTITGKCLYGQ-PVFRKVHAREHPIPSTVITITEECDDKQPLT
Sloth_NCX                   LNELGG-FTITGKYLYGQ-PVFRKVHARERLIPSTVINFAEEFDDKQPLT
Hyrax_NCX                   XNELGD-FTITGKYLYGQ-PVFRKVHAREHPIPSTVINFSDEYEEKQPLT
Opossum_NCX                 LNELGD-FTLTGSFLLDQ-PVFRKVHVRERPLPSTIINFAEECDDKQPLT
Platypus_NCX                -----------GGKLLQN----------------------EANDKQPLT
Mouse_NCX                   LNELGG-FTLTGKEMYGQ-PIFRKVHARDHPIPSTVITISEEYDDKQPLT
Kangaroo_rat_NCX            LNELGG-FTLTGKYLYGQ-PIFRKVLAREHPIPSTVITIAEEGDVKKPLT
Turkey_NCX                  LNELGG-FTITGGKLWKGKPVFRKVQARERPLPCTVVTIREENEEKQPLT
Zebra_finch_NCX             LNELGG-FTITGMDLYSS-PVPLKVQARDHPLPCTVVSIQEENEEKQPLT
Chicken_NCX                 ------------------------VWLRRG--------VKEENEEKQPLT
Xenopus_tropicalis_NCX      LNELGD-FTITGKILYGK-PVLRKVQVRDHPIPSTVIILTEENEEKQPLT
Stickleback_NCX             LQEVGG-FVKTGR------DVYRKVQGRDHPAPSAVISITDEGGEEV-LT
Zebrafish_NCX               LHECGG-FVKTGR------DVYRKVQGRDKPIPSTIISISEDGEEET-LT
C.elegans_NCX               ---------------------------------------------HRELA
C.intestinalis_NCX          -SVFPFRVEYNNSYSKRV---KFWVELADPYSPNNDSGINGKEIDKKATE
C.savigny_NCX               TSSLEPWVPGRDVLRKDK-------DPSPQIYAGSDSGINGKEDEKKKTD
drosophila_NCX              -------------------------ELAAKI----------KEVEKKPVQ
                                                                         .

                          655
Canine_NCX1                 SKEEEERRIAEMGRPILGEHTKLEVIIEESY
Medaka_NCX                  GPED----VSKMGCPILGEHTKLEVVIEESY
Tetraodon_NCX               GPED----VSKMGCPCLGEHTKLEVVIEESY
Fugu_NCX                    GPED----VSKMGCPSLGEHTKLEVVIEESY
Anole_Lizard_NCX            GGEE----IAKMGCPTLGEYTKLEVIIEESY
Human_NCX1                  SKEEEERRIAEMGRPILGEHTKLEVIIEESY
Rat_NCX                     SKEEEERRIAEMGRPILGEHTKLEVIIEESY
Megabat_NCX                 SKEEEERRIAEMGRPILGEHTKLEVIIEESY
Tarsier_NCX                 SKEEEERRIAEMGRPILGEHTKLEVIIEESY
Alpaca_NCX                  SKEEEERRIAEMGRPILGEHTRLEVIIEESY
Cow_NCX                     SKEEEERRIAEMGRPILGEHTRLEVIIEESY
Dolphin_NCX                 SKEEEERRIAEMGRPILGEHTRLEVIIEESY
Chimpanze_NCX               SKEEEERRIAEMGRPILGEHTKLEVIIEESY
Gorilla_NCX                 SKEEEERRIAEMGRPILGEHTKLEVIIEESY
Orangutan_NCX               SKEEEERRIAEMGRPILGEHTKLEVIIEESY
Gibbon_NCX                  SKEEEERRIAEMGRPILGEHTKLEVIIEESY
Macaque_NCX                 SKEEEERRIAEMGRPILGEHTKLEVIIEESY
Marmoset_NCX                SKEEEERRIAEMGRPILGEHTKLEVIIEESY
Guinea_pig_NCX              SKEEEERRIAELGRPILGEHTKLEVIIEESY
Pika_NCX                    SKEEEERRIAEMGRPILGEHTKLEVIIEESY
Bushbaby_NCX                SKEEEERRIAEMGRPILGEHTKLEVIIEESY
Microbat_NCX                SKEEEERRIAEMGRPILGEHTKLEVIIEESY
Sloth_NCX                   SKEEEERRIAEMGRPILGEHTKLEVIIEESY
Hyrax_NCX                   SKEEEERRIAEMGRPILGEHTRLEVIIEESY
Opossum_NCX                 SKEEEERRIAEMGRPILGEHTKLEVIIEESY
Platypus_NCX                SKEEEERRIAELGRPILGEHTKLEIIIEESY
Mouse_NCX                   SKEEEERRIAEMGRPILGEHTKLEVIIEESY
Kangaroo_rat_NCX            SKEEEKRRIAELGRPILGEHTRLEVIIEESY
Turkey_NCX                  SKEEEERRIAEMGRPVLGEHTKLEVIIEESY
Zebra_finch_NCX             SKEEEERRIAEMGRPVLGEHTKLEIIIEESY
Chicken_NCX                 SKEEEERRIAEMGRPVLGEHTKLEVIIEESY
Xenopus_tropicalis_NCX      SKEEEERRIAEMGRPVLGEHTRLEIIIEESY
Stickleback_NCX             KKEKEERRIAEMGRPMLGEHIKLEVIIEESY
Zebrafish_NCX               KKEKDERRIAEMGRPTLGEHVKLEVIIEESY
C.elegans_NCX               DDEE-----GIEGKPILG-FSRCKVVITEDR
C.intestinalis_NCX          E-EEEARRIAELGKPRLGAETTVEIIIEESY
C.savigny_NCX               EQEEEARRIAELGKPRLGAQTSIEVMIEESY
drosophila_NCX              D-LTELDRILLLSKPRNGELTTAYVRIRESQ
                                        . *  *      : * *.
```
