# Supplementary material for: A Common Ca2+-Driven Interdomain Module Governs Eukaryotic NCX Regulation
Source: PLoS One. 2012 Jun 29;7(6):e39985. doi: 10.1371/journal.pone.0039985 (PMC3386913; doi:10.1371/journal.pone.0039985)
Supplement: Table S1 — CBD12 Interface Conservation. (DOCX) [file pone.0039985.s004.docx]

| **Residue** | **Conservation Score** | **B-factor (Å^2^)** |
| --- | --- | --- |
| N386 | -1.149 | 78.4 |
| T415 | -0.999 | 53.5 |
| N417 | -0.533 | 68.6 |
| D448 | -1.121 | 81.2 |
| I449 | -0.981 | 48.1 |
| F450 | -0.757 | 37.8 |
| E451 | -1.106 | 51.8 |
| E452 | -0.896 | 63.3 |
| E454 | -1.106 | 43.8 |
| D499 | -1.121 | 72.5 |
| D500 | -1.121 | 55.1 |
| H501 | -1.135 | 45.3 |
| A502 | -1.146 | 38.3 |
| G530 | -1.046 | 47.6 |
| A531 | -1.146 | 59.9 |
| R532 | -1.12 | 59.5 |
| D565 | -0.725 | 82.6 |
| K621 | 0.547 | 104.1 |
| E624 | -0.732 | 69.9 |
| E625 | 0.175 | 73.3 |
| I628 | -0.929 | 47.6 |
| A629 | -0.464 | 48 |
| M631 | 0.163 | 69.7 |
| G632 | -0.774 | 52.2 |
