# Supplementary material for: A Common Ca2+-Driven Interdomain Module Governs Eukaryotic NCX Regulation
Source: PLoS One. 2012 Jun 29;7(6):e39985. doi: 10.1371/journal.pone.0039985 (PMC3386913; doi:10.1371/journal.pone.0039985)
Supplement: Table S2 — Overall SAXS parameters for CBD12 and its mutants. R g, D max, and NSD are, respectively, the radius of gyration derived from Guinier plotting, maximum inter-atomic dimension, normalized shape discrepancy for DAMMIN calculation. Note that NSD scores for all bead models are less than 0.8, indicating high convergence of the bead model calculations. (DOCX) [file pone.0039985.s005.docx]

| Sample | *R*_g_ | *D*_max_ | NSD |
| --- | --- | --- | --- |
| CBD12 with EDTA | 34.9±0.6 | 130±1 | 0.70±0.03 |
| CBD12 with Ca^2+^ | 32.6±0.5 | 107±1 | 0.70±0.03 |
| 7A with EDTA | 34.3±0.6 | 133±1 | 0.66±0.02 |
| 7A with Ca^2+^ | 34.8±0.7 | 130±2 | 0.70±0.05 |
| R532A with EDTA | 34.9±0.3 | 135±2 | 0.64±0.04 |
| R532A with Ca^2+^ | 35.2±0.5 | 135±2 | 0.69±0.02 |
| E454K with EDTA | 29.6±0.4 | 103±2 | 0.79±0.02 |
| E454K with Ca^2+^ | 31.8±0.3 | 105±2 | 0.79±0.02 |
|  |  |  |  |
|  |  |  |  |
