# Supplementary material for: A Common Ca2+-Driven Interdomain Module Governs Eukaryotic NCX Regulation
Source: PLoS One. 2012 Jun 29;7(6):e39985. doi: 10.1371/journal.pone.0039985 (PMC3386913; doi:10.1371/journal.pone.0039985)
Supplement: Information S1 — Experimental procedures. (DOCX) [file pone.0039985.s006.docx]

**Supplementary Information**

**Experimental Procedures**

**SAXS data collection, analysis and bead model reconstruction.** For the data recorded at the APS, the energy of the X-ray beam was 12 KeV (wavelength λ=1.033 Å), and the distance from the sample to detector (PILATUS 2M, Dectris Ltd) was 2 meters, covering a scattering vector range (q=4πsinθ/λ) from 0.007 to 0.66 Å^-1^, as determined by the scattering profile of silver behenate. 20 frames of two-dimensional images were recorded for each buffer or sample using a flow cell, with the exposure time of 5s for each frame to improve signal/noise ratio and with a sleep time of 1 second between each frame to reduce radiation damage. For the data recorded at the ESRF, the energy of the X-ray beam was 13.32 KeV (wavelength λ=0.931 Å), and the distance from the sample to detector (PILATUS 1M, Dectris Ltd) was 2.43 meters, covering a scattering vector range from 0.005 to 0.5 Å ^-1^. 10 frames of two-dimensional images were recorded for each buffer or sample using a flow cell, with the exposure time of 10 s for each frame. The 2D images were reduced to one-dimensional scattering profiles using the Matlab scripts on site. The scattering profile of a sample solute was calculated by subtracting the buffer contribution from the sample-buffer profile using the program PRIMUS (1). The experimental radius of gyration (*R*_g_) and the forward scattering intensity *I*(0) were calculated from data at low *q* values in the range of *qR*_g_ < 1.3, using the Guinier approximation: ln*I*(*q*)≈ln(*I*(0))-*R*_g_^2^*q*^2^/3. The pair distance distribution function (PDDF), p(r), and the maximum dimension of the protein, *D*_max_, in real space were calculated with the indirect Fourier transform using the program GNOM (2). To avoid underestimation of the molecular dimension and consequent distortion in low resolution structural reconstruction, the parameter *D*_max_, the upper end of distance *r*, was chosen such that the resulting PDDF has a short, near zero-values tail at large *r*. Ab initio shape reconstructions were performed with the programs DAMMIN ([3](#_ENREF_5)), using scattering data of the q range between 0.02 and 0.30 Å^-1^. 32 independent DAMMIN calculations for each construct were performed, The resulting bead models were subjected to averaging by DAMAVER (4) where the normalized spatial discrepancy (NSD) values between each pair of models were computed. The model with lowest average NSD with respect to the rest of models was chosen as the reference model. The remaining models were superimposed onto the reference model using SUPCOMB (5) except that possible outliers identified by NSD criteria were discarded. The dummy atoms of these superimposed models were remapped onto a densely packed grid of atoms with each grid point marked with its occupancy factor. The grids with non-zero occupancies were chosen to generate a final consensus model with the volume equal to the average excluded volume of all the models. The final models were filtered using DAMFILT.

**References**

1. Konarev, P.V., Volkov, V.V., Sokolova, A.V., Koch, M.H.J, Svergun, D.I. (2003) *J Appl Cryst* **36**, 1277-1282.

2. Svergun, D.I. (1992) J Appl Cryst **25**, 495-503

3. Svergun, D. I. (1999) *Biophys J* **76**, 2879-2886

4. Volkov, V.V., Svergun, D.I. (2003) *J Appl Cryst* **36**, 860-864.

5. Kozin, M., Svergun, D.I. (2001) *J Appl Cryst* 34:33-41.
